# Probiotic Ameliorating Effects of Altered GABA/Glutamate Signaling in a Rodent Model of Autism

**DOI:** 10.3390/metabo12080720

**Published:** 2022-08-04

**Authors:** Rawan M. Bin-Khattaf, Mona A. Alonazi, Abeer M. Al-Dbass, Ahmad T. Almnaizel, Hisham S. Aloudah, Dina A. Soliman, Afaf K. El-Ansary

**Affiliations:** 1 Biochemistry Department, Science College, King Saud University, P.O. Box 22452, Riyadh 11495, Saudi Arabia; 2 Experimental Surgery and Animal Lab, College of Medicine, King Saud University, P.O. Box 2925, Riyadh 11461, Saudi Arabia; 3 Department of Botany and Microbiology, Science College, King Saud University, P.O. Box 22452, Riyadh 11495, Saudi Arabia; 4 Central Research Laboratory, Female Campus, King Saud University, P.O. Box 22452, Riyadh 11495, Saudi Arabia

**Keywords:** autism spectrum disorders, propionic acid, oxidative stress, glutamate excitotoxicity, γ-aminobutyric acid, GABA receptors, gene expression

## Abstract

Autism spectrum disorders (ASDs) comprise a heterogeneous group of pathological conditions, mainly of genetic origin, characterized by stereotyped behavior, such as marked impairment in verbal and nonverbal communication, social skills, and cognition. Excitatory/inhibitory (E/I) imbalances have been recorded as an etiological mechanism of ASD. Furthermore, GABA, the main inhibitory neurotransmitter in adult life, is known to be much lower in both patients and rodent models of ASD. We propose correcting GABA signaling as a therapeutic strategy for ASD. In this study, 40 young male western Albino rats, 3–4 weeks in age, weighing about 60–70 g, were used. The animals were randomly assigned into six experimental groups, each including eight rats. Group I served as the control group and was orally administered phosphate-buffered saline. Groups II and III served as rodent models of ASD and were orally administered a neurotoxic dose of propionic acid (PPA). The rats in the three therapeutic groups (IV, V, and IV) received the same doses of PPA, followed by 0.2 g/kg body weight of pure *Bifidobacterium infantis*, a probiotic mixture of ProtexinR, and pure *Lactobacillus bulgaricus*, respectively, for 3 weeks. Selected variables related to oxidative stress, glutamate excitotoxicity, and gut bacteria were measured in the six groups. Both pure and mixed *Lactobacillus* and *Bifidobacterium* were effective in ameliorating glutamate excitotoxicity as an autistic feature developed in the PPA-induced rodent model. Their therapeutic effects mostly involved the correction of oxidative stress, restoration of depleted GABA, and up-regulation of GABA receptor gene expression. Pure *Bifidobacterium* was the most effective, followed by the mixture of probiotics and finally * lactobacillus*. In conclusion, *Bifidobacteria* and *lactobacilli* can be used independently or in combination as psychobiotics to ameliorate oxidative stress and glutamate excitotoxicity as two confirmed etiological mechanisms through the gut–brain axis.

## 1. Introduction

One of the etiologies of neurodevelopmental disorders is imbalanced neurotransmitter signaling. Various associated proteins, such as receptors and transporters of the neurotransmitters, are usually involved in the clinical presentation of many neurological disorders, such as autism spectrum disorders (ASDs). For example, γ-aminobutyric acid (GABA), glutamate (Glu), serotonin (5-HT), and dopamine (DA) are related to deficits in autism, a type of ASD. Ref. [1] Glutamatergic/GABAergic imbalance can be found in ASD and anxiety disorders, with elevated glutamatergic neurotransmission as an excitatory neurotransmitter, concomitant with lower GABA as inhibitory neurotransmission [2,3]. Glutamate increase is responsible for excitotoxicity, leading to neuronal injury, cell death, and dysfunction of surviving neurons; however, delayed disruption of excitatory glutamate circuits leads to deficits in cognitive and motor function and in experience-dependent plasticity. GABA modulates excitatory pathways in the brain, and following injury, the loss of GABA-producing cells disrupts the balance of excitation and inhibition, leading to further cell injury and apoptosis [4].

The brain–gut–microbiota axis is a bidirectional communication system enabling crosstalk between the brain and the gut microbiota [5]. It is composed of the central nervous system (CNS, represented by the brain), the enteric nervous system, and the digestive system [6]. This confirms that the gut microbiota can directly affect the GABAergic system by regulating the synthesis and release of key GABA from bacteria. Lactobacillus and Bifidobacterium species can reportedly produce GABA. These microbially synthesized GABA can cross the mucosal layer of the intestines and indirectly affect brain functions, acting on the enteric nervous system and modulating immune system activation. Altered gut microbiota have been consistently documented in autistic individuals, with much higher growth of Clostridium spp. as propionobacteria [7,8]. Manipulating the gut microbiota with probiotics and prebiotics could be a novel approach to treating neurodevelopmental disorders through the gut–brain axis [9].

It is well documented that PPA, usually generated by overgrown bacteria, such as clostridial and others, as a metabolite, is successfully used to induce persistent autistic features in rodents [7].

In the bidirectional communication of the gut–brain axis, Lactobacillus and Bifidobacterium strains are recognized to be useful therapeutic adjuncts in multiple neurological disorders [10,11]. This information led us to test the effect of pure Lactobacillus, pure Bifidobacterium, and a mixture of probiotics in ameliorating selected variables related to oxidative stress, GABA/glutamate signaling, and gut microbiota and suggest the use of probiotics as an early intervention strategy in ASD.

## 2. Results and Discussion

**Figure 1:**
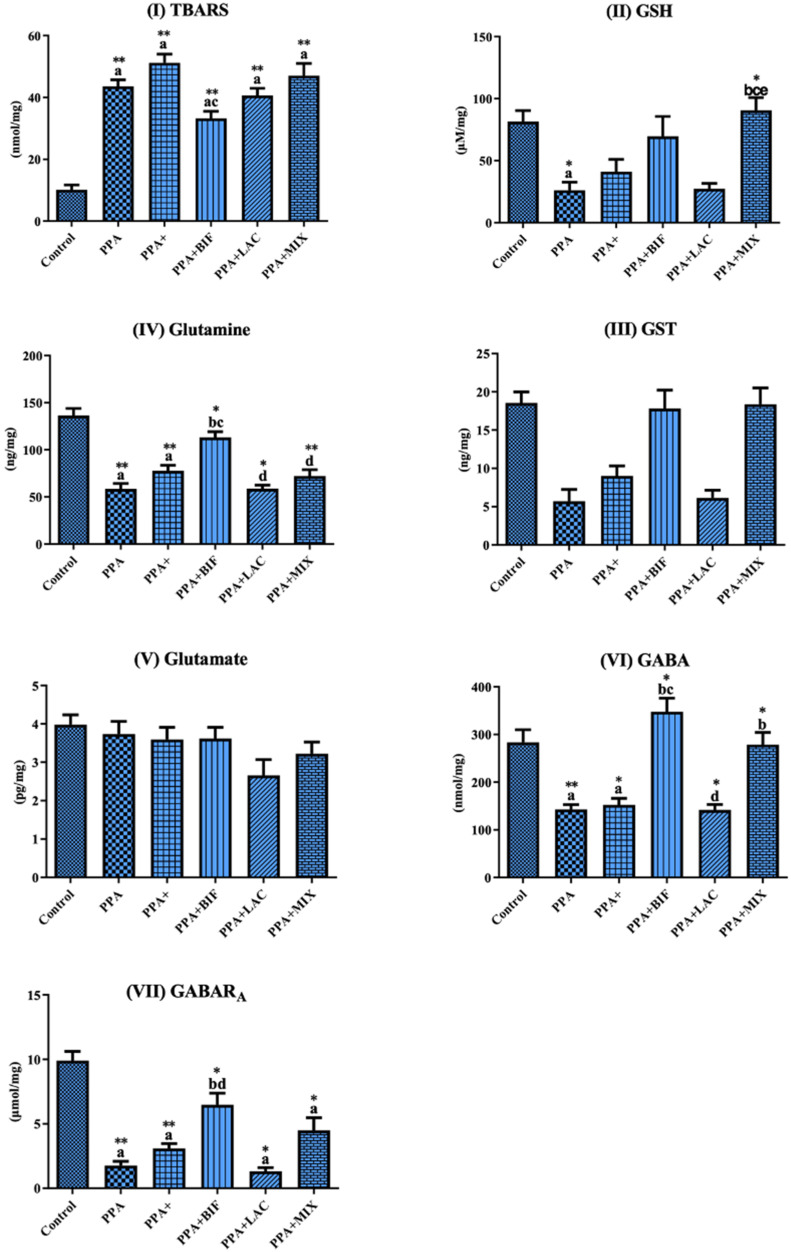
Concentrations of (**I**) TBARS, (**II**) GSH, (**III**) GST, (**IV**) glutamine, (**V**) glutamate, (**VI**) GABA, and (**VII**) GABAR_A_ in the brain homogenates of male western albino young rats in all groups. (a) Control vs. all groups, (b) PPA vs. all groups, (c) PPA + vs. therapeutic groups, (d) PPA + BIF vs. PPA + LAC and PPA + BIF vs. PPA + MIX, and (e) PPA + LAC vs. PPA + MIX. (*) The mean difference is significant at *p* ˂ 0.001, and (**) the mean difference is significant at *p* ˂ 0.0001.

**Figure 2:**
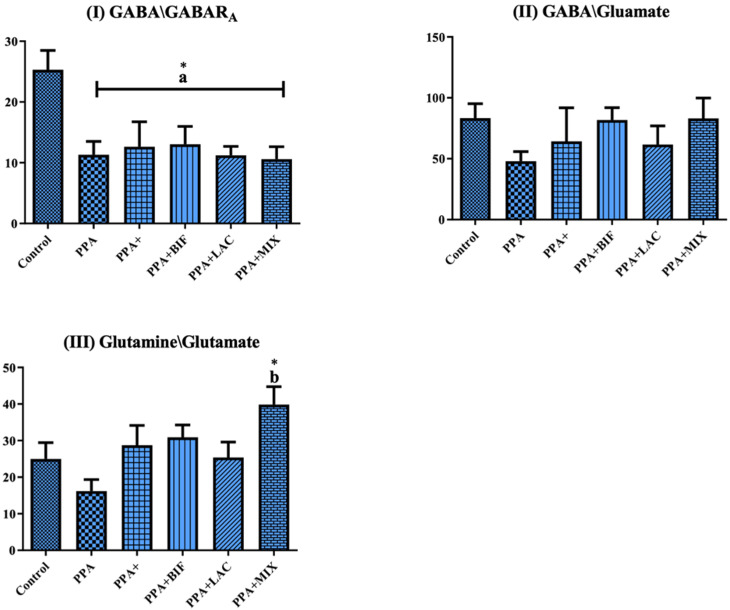
Ratios of (**I**) GABA/GABAR_A_, (**II**) GABA/glutamate, and (**III**) glutamine/glutamate in the brain homogenates of male western albino young rats in all groups. (a) Control vs. all groups and (b) PPA vs. all groups. (*) The mean difference is significant at *p* ˂ 0.001.

**Figure 3:**
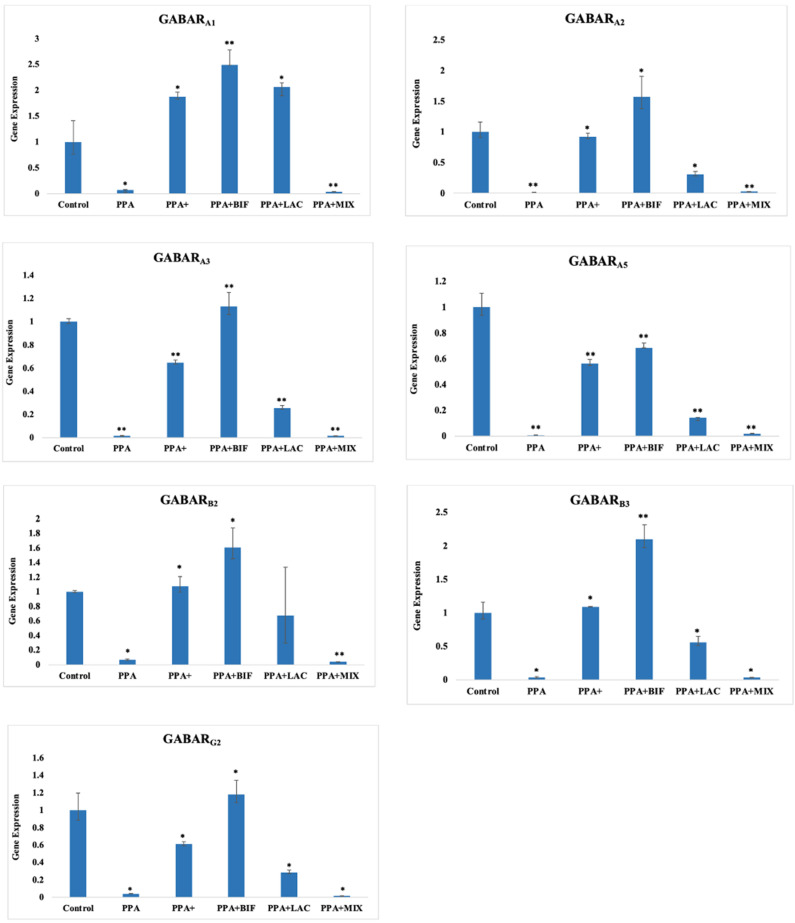
Effect of probiotic treatments on the gene expression of GABAR_A_, GABAR_B_, and GABAR_G_ selected subunits in the brain homogenates of male western albino young rats in all groups. (*) The mean difference is significant at *p* ˂ 0.001, and (**) the mean difference is significant at *p* ˂ 0.0001.

To determine if the use of probiotics could be effective in ameliorating the altered GABA/glutamate signaling in the rodent model of ASD, selected biomarkers related to oxidative stress and GABA signaling were measured in brain homogenates of control (Group I), PPA-treated rats as a rodent model of autism (Groups II and III), the three therapeutic groups (IV, V, and VI) received the same doses of PPA, followed by 0.2 g/kg body weight of probiotic (ProtexinR), healthy bacteria Bifidobacterium infantis, and healthy bacteria Lactobacillus bulgaricus, respectively, for 3 weeks. The results are expressed as means ± SD. Table 1 and Figure 1 display (1) TBARS as a biomarker of oxidative stress that significantly increases; the significant decrease in GSH and GST as markers of antioxidant status; and lower glutamine, GABA, and GABARA as markers related to GABA signaling in PPA-treated rats as a rodent model of ASD and (2) the ameliorative effects of the used probiotics, with Bifidobacterium supplement being the most effective at *p* < 0.0001. Table 2 and Figure 2 demonstrate the relative GABA/GABARA, GABA/glutamate, and glutamine/glutamate ratios in the six studied groups. GABA/GABARA was significantly lower in the six groups relative to the control group, while the other two ratios were significantly reduced in PPA-treated rats and remarkably corrected in animals treated therapeutically with probiotics. Table 3 and Figure 3 demonstrate the significant increase in different GABA receptor gene expression in ProtexinR, Bifidobacterium infantis, and Lactobacillus bulgaricus-treated groups at *p* < 0.0001 or 0.001. Table 4 presents the differences in microorganisms in the six studied groups at the end of the study. The decrease in Moraxella as lactate producer in PPA-treated rats compared to the control group and the ameliorative effect of the three probiotics used are easily noticed.

Oxidative stress is one of the topmost penalties of glutamate-induced neurotoxicity. However, it is not possible to describe a unidirectional cause–effect connection between the two signaling as etiological mechanisms of ASD since oxidative stress and excessive intracellular ROS can also induce excitotoxicity by inducing the extracellular discharge of glutamate [12] and releasing calcium from mitochondria into the cytosol [13]. From another perspective, it has already been shown that astrocytic glutamine synthetase is especially susceptible to ROS-induced inactivation, which negatively affects the complete glutamate–glutamine cycle and contributes to an increase in extracellular glutamate concentrations, resulting in excitotoxicity [14]. Additionally, the presence of ROS has been shown to decrease the activity of glutamate transporter, weakening synaptic clearance of glutamate and further contributing to increased extracellular glutamate levels [15]. Collectively, this could support the remarkable increase in lipid peroxides concomitant with the decrease in glutathione, GST, glutamine, and glutamine/glutamate ratios in PPA-treated groups as a rodent model of ASD (Table 1 and Figure 1I–V; Table 2 and Figure 2III). This is in good agreement with numerous previous works that have demonstrated alteration in multiple biomarkers related to oxidative stress and glutamate excitotoxicity in a PPA-induced rodent model of autism [16,17,18]. Lower glutamine could lead to a much higher toxic effect of glutamate as an excitatory neurotransmitter, while lower GABA and GABA receptors could be easily related to the E/I imbalance repeatedly reported in autistic patients and rodent models [19,20]. Reversal of GABAergic/glutamatergic imbalance was recently reported as a medical hypothesis to treat ASD [3]. Among the integrated strategies to reverse E/I imbalance is the use of probiotics as a source of GABA and inducer of GABA receptor gene expression.

Table 1 and Figure 1I–V and Table 2 and Figure 2III also demonstrate the significant improvement in lipid peroxides, glutathione, GST, glutamine, and glutamine/glutamate ratios in probiotic-treated rats. This is in good agreement with a previous work by Duranti et al. [21], in which they reported a slight decrease in glutamate concentration after the growth of *B. dentium*. While glutamate levels were somewhat unchanged in their study, which is in good agreement with this study, they observed increased concentrations of GABA and glutamine in *B. dentium*-conditioned fully defined bacterial media, called ZMB1, for 18 h.

GABAergic neurons produce GABA from glutamate using glutamic acid decarboxylase (GAD), and this synthesized GABA is packaged into synaptic vesicles at synaptic terminals through vesicular GABA transporters (VGATs). Synaptically released GABA binds to both presynaptic and postsynaptic GABA receptors (GABAA and GABAB) and suppresses the excitation as a neurotoxic mechanism repeatedly reported in ASD [3,17].

Table 1 and Figure 1VI,VII also demonstrate the significant increase in GABA and GABA receptors in probiotic-treated rats, with *Bifidobacterium* strains being the most effective, followed by the probiotic mixture, while *lactobacillus* was the least effective. This finds support in the work of Yunes et al. [10], in which they screened the ability of 135 human-derived *lactobacillus* and *bifidobacterium* strains to produce GABA from glutamate. They reported that the most efficient GABA producers were *Bifidobacterium* strains, a finding in good agreement with the results of this study. Their data indicate that *gad* genes, as well as the ability to produce GABA, are widely distributed among *lactobacilli* and *bifidobacteria*, which could help in the use of both bacterial strains independently or in combination as psychobiotics [10]. In a recent study by Duranti et al. [21], in Groningen rats fed on a diet supplemented with *B. adolescentis* strains, the in vivo production of GABA was stimulated, highlighting their potential implication in gut–brain axis interactions. On the basis of the significant role of PPA in decreasing PTEN (Akt inhibitor), leading to gliosis, disturbed neurocircuitry, and inflammatory response as seen in ASD, the therapeutic effects of the used probiotics could be attributed to the up-regulation of PTEN signaling, down-regulation of Akt genes, and restoration of GABA A receptor function [22,23]. Going by all these studies, we can suggest that probiotics have demonstrated an effect in the form of mono- and multi-strains and could help treat infectious diarrhea, antibiotic-associated diarrhea, and diarrhea associated with *Clostridium difficile*, a problem related to GI co-morbidity in ASD patients [24,25,26].

Balanced neural circuits are especially important for proper social and emotional behavior, language processing, and higher-order cognition. Research in this area has shown that alterations in GABAergic signaling inhibition activities could result in a loss of balance in neural circuits and lead to a disproportionately high level of excitation. Importantly, evidence accumulated from previous studies indicates that disruption in GABAergic signaling transmission could potentially lead to the development of autism [27,28].

Specific ratios between selected pairs of metabolite concentrations (metabolite ratios) have been introduced in the past as biomarkers in many biomedical applications [26]. Glutamate/GABA and glutamate/ glutamine ratios were used to evaluate the biochemical and functional relationship of impaired glutamate signaling in ASD patients, and both recorded much higher predictive values [28,29,30].

Table 2 and Figure 2I–III demonstrate the relative ratios of GABA/GABAAa, GABA/glutamate, and glutamine/glutamate. These three ratios established the impaired glutamate signaling in response to PPA-induced neurotoxicity in the rodent model. In addition, the remarkable ameliorative effects of the three probiotic interventions used were established, noticeably seen as much higher GABA/glutamate and glutamine/glutamate ratios. As previously discussed, this is in good agreement with multiple clinical or experimental works on ASD [15,16,17].

The significant decrease in or downregulation of all the seven studied GABA receptor subunits in PPA-treated rats supports the validity of this model and explains the recently recorded alteration in social interaction as an autistic feature in male rats treated with PPA [7]. With lower and dysfunctional GABA receptors, an imbalanced E/I signal can be induced as an autistic feature (Table 3 and Figure 3). The three probiotic intervention strategies used clearly demonstrate significant up-regulation of the studied GABA receptor subunit gene expression, with pure *Bifidobacterium* strains being the most potent, followed by the probiotic mixture, while *lactobacillus* was the least effective. This is supported by the work of Bravo et al. [11], which proves the effectiveness of probiotics in up-regulating GABA receptor subunits and highlights the possible mechanistic vision into the potential effect of probiotics in treating anxiety-like behavior [31,32].

Table 4 demonstrates the changes in microorganisms in the six studied groups at the end of the study. The decrease in lactate producers in PPA-treated rats compared to the control group and the ameliorative effect of the three probiotics used are easily observed. This result is seemingly supported by the study of Kang and colleagues [33], which recorded a much lower growth rate of certain species of lactate fermenters in subjects with autism [34,35]. The decrease can partly explain the high levels of lactate observed in autistic patients [36]. There was a noticeable increase in *Staphylococcus* and *Moraxella* species in probiotic-treated rats compared to those in the PPA-induced model, which is in good agreement with the recent works of Forsyth [28] and Al-Dera [37], which reported a decrease in the abundance of certain bacteria, for example, *Moraxella* in autistic individuals and the PPA-rodent model of ASD. Lower *Moraxella* in the PPA-rodent model was attributed to a leaky gut as an accepted phenomenon relating gut microbiota to brain disorders through the gut–brain axis.

The remarkable increase in *Moraxella* in the probiotic-treated groups finds support in the work of van den Broek et al. [38], which recorded the inhibitory effect of lactobacilli against the respiratory tract pathogen *Moraxella catarrhalis.*

Since a lower GABA/glutamate ratio, altered gut microbiota, and overgrowth of propionobacteria are promising targets for a precision medicine approach to ASD treatment [39,40], the ameliorating effects of the tested probiotics displayed in this study suggest measuring the GABA/glutamate ratio by magnetic resonance spectroscopy (MRS) as a neuroimaging biomarker for assessing the treatment efficacy of probiotics for ASD patients. This finds support in a recent randomized clinical trial that proved potentially positive effects of probiotics on core autistic symptoms in a subgroup of ASD children independent of the specific intermediation of the probiotic effect on GI symptoms [41].

Although the cause–effect connection between ASD and gut microbiota is not yet well proven, the anti-depressive effects, improvement in mental health, and gut microbiota composition of stressed individuals after consuming *Lactobacillus* and *Bifidobacterium* populations indicate the importance of the gut–brain axis as a target to treat ASD [42].

Supplementation of specific probiotics may represent a side-effect-free tool to re-establish gut homeostasis and promote brain health through the gut–brain axis. Thus, pure *Bifidobacterium infantis*, the probiotic mixture of ProtexinR, and pure *Lactobacillus bulgaricus* could be recommended as a non-pharmacological adjuvant therapy for children with ASD that could be accessed at home.

## 3. Materials and Methods:

### 3.1. Animals

Forty young male western Albino rats, 3–4 weeks in age, weighing about 60–70 g, obtained from the experimental surgery and animal laboratory, were enrolled in this study. The animals were randomly assigned into six experimental groups, each including eight rats. The animals in the control group (I) were orally administered phosphate-buffered saline. The animals in the PPA-treated groups (II and III) were orally administered a neurotoxic dose of PPA (250 mg/kg body weight/day) for 3 days. Group II was euthanized after 3 days, while Group III was kept alive to be euthanized with other groups [16]. The rats in the three therapeutic groups (IV, V, and VI) received the same doses of PPA, followed by 0.2 g/kg body weight of probiotic (ProtexinR), healthy bacteria *Bifidobacterium infantis*, and healthy bacteria *Lactobacillus bulgaricus*, respectively, for 3 weeks. ProtexinR (Somerset, UK) is a mixture of some healthy bacteria, such as *Bifidobacterium infantis*, *Bifidobacterium breve*, *Lactobacillus acidophilus*, *Lactobacillus bulgaricus*, *Lactobacillus casei*, *Lactobacillus rhamnosus*, and *Streptococcus thermophiles*, with the concentration of 1 billion CFU per gram.

The rats were placed at 22 ± 1 °C with ad libitum access to water and standard chow, and quantitative stool cultures were carried out. The experiment protocol was in accordance with the ethical standards of the ethics committee responsible for animal experimentation at King Saud University, Riyadh, and was approved according to the Helsinki Declaration of 1975, as revised in 2008 (http://www.wma.net/en/20activities/10ethics/10helsinki/, accessed on 18 June 2022). (IRB NO.: KSU-SE-19-131).

### 3.2. Preparation of Brain Tissue Homogenates

By the end of the feeding trials, deeply anesthetized (by using Ketamine/Xylazine + D.W (91, respectively 9 mg/kgbw, I.P.) rats were decapitated. The brain tissues were retrieved from the rats in the six groups and dissected into small pieces, homogenized in bi-distilled water (1:10, *w/v*), and stored at −30 °C until further use.

### 3.3. Biochemical Analyses

#### 3.3.1. ELISA Measurements of GABA-Signaling-Related Markers

The enzyme-linked immunosorbent assay (ELISA) technique was used to measure the parameters in the brain tissue homogenate from all the experimental animal groups. The applied assays were based on the method of sandwich or competitive binding enzyme immunoassay technique. While the sandwich ELISA principle was used to estimate Gamma-Aminobutyric Acid Receptor Subunit Alpha-1 (GABRA1) and glutamate, competitive ELISAs were used to estimate GABA and glutamine markers. All tests were quantitatively determined according to the manufacturer’s instructions using ELISA kits from MyBioSource (San Diego, CA, USA). The following markers were estimated: GABRA1 (Cat No.: MBS9342109), with a detection range of 0.5–16 umol/L; GABA (Cat No. MBS269152), with a detection limit of 2000–31.2 pg/mL; glutamate (Cat No. MBS 269969), with a sensitivity of 1.0 ng/mL; glutamine (Cat No.: MBS755884), with a sensitivity of 1.0 ng/mL; and Glutathione S-Transferase (Cat No.: MBS564158).

#### 3.3.2. Measurement of Lipid Peroxidation Concentration

The method of Potter et al. [43] was used for lipid oxidation, which is estimated by the formation of thiobarbituric-acid-reactive substances.

#### 3.3.3. Assay of Glutathione (GSH)

The GSH content was determined according to the method described by Beutler et al. [44] using 5,5′-dithiobis 2-nitrobenzoic acid (DTNB) with sulfhydryl compounds to produce a relatively stable yellow color.

### 3.4. Gene Expression

The gene expression of GABA in brain tissue was determined according to the method described in [45]. Total RNA was purified from the rat brain tissue using the RNAeasy^®^ Lipid Tissue Mini Kit (Qiagen, Germany) (Cat No. 74104). To prepare complementary DNA (cDNA), purified total RNA from each sample was reverse-transcribed by random hexamers of the high-capacity cDNA Reverse Transcription kit (Applied Biosystems, Foster City, CA, USA). GABAergic expression was estimated by quantitative RT-PCR (Light Cycler 480 II/96, Roche Applied Science, Basel, Basel-Stadt, Switzerland) using an iTaq™ Universal SYBR^®^ Green Super mix kit prepared according to the manufacturer’s protocol Gene Expression Assay (Assay ID Rn00691548_m1, Applied Biosystems). Gene expressions of glyceraldehyde-3-phosphate dehydrogenase (GAPDH) were used as a reference gene (assay ID Rn01775763g1, Applied Biosystems). Specific primers were added to the reaction mix at a final concentration of 10 pM.

### 3.5. Microbial Analysis

#### 3.5.1. Fecal Collection and Preparation for Microbial Analysis

The fecal samples of the rats from all groups were collected in sterile tubes and kept at −20 °C until further use. The microbial analysis involved culturing microorganisms on different media and under different incubation conditions for their preliminary identification and enumeration to identify the alteration in the gut microbiota in response to the treatment being tested.

Fecal suspensions of each treated group correspondingly were prepared by dissolving 1:10 w/v in sterile phosphate-buffered saline (PBS, 0.1 M) [46].

All samples were homogenized using a sonicator for 5 s and then centrifuged at 5000 rpm for 10 min at −4 °C. Ten-fold serial dilution of the fecal suspensions was then performed. To begin with, 1 mL of the supernatant from the original dilution (dilution 0) was added to 9 mL of sterile PBS in a tube (dilution 1). The process was repeated until dilution 4 was created, and 0.1 mL of each of the prepared dilutions was loaded on the surface of different culture media and spread. The culture media used included the nutrient agar and were incubated aerobically at 37 °C for 18–24 h.

#### 3.5.2. Bacterial Enumeration and Identification

Before incubation, the bacterial count from the different media was recorded as the colony count per plate. Data of the rat groups used in the study were compared. Preliminary bacterial identification was performed by morphological observation on the different media used. Further identification was made microscopically using the Gram-staining technique, where single colonies from the various culture media were selected, heated to form a smear, subjected to a Gram-staining procedure, and then observed under the microscope using an oil immersion lens.

#### 3.5.3. Statistical Analyses

The results of this study were expressed as the means ± SD. All statistical comparisons between the control group and the PA and probiotic-treated rat groups were performed using SPSS V, 21 software package (SPSS Inc., Chicago, IL, USA). One-way analysis of variance (ANOVA) tests with Dunnett’s test for multiple comparisons was performed.

## 4. Conclusions

The study indicates that pure or mixed lactobacillus and *bifidobacterium* can be used to ameliorate glutamate excitotoxicity induced in a rodent model of autism, mostly through the amelioration of oxidative stress, increasing the depleted GABA and up-regulating the gene expression of GABA receptors. Collectively, these data highlight the important role of probiotics in the bidirectional communication of the gut–brain axis and suggest that certain organisms may prove to be useful in therapy.

## Figures and Tables

**Table 1:** Means ± SD of all the measured variables in the brain homogenates of the six studied groups.

	Control	PPA	PPA+	PPA + BIF	PPA + LAC	PPA + MIX
**TBARS**	10.17 ± 3.528	43.61 ± 5.181 a**	51.24 ± 6.843 a**	33.30 ± 6.274 ac**	40.68 ± 6.111 a**	47.09 ± 10.41 a**
**GSH**	81.62 ± 19.59	26.21 ± 15.94 a*	41.16 ± 24.21	69.70 ± 45.15	27.39 ± 11.51	90.67 ± 27.27 bce*
**GST**	18.55 ± 3.219	5.690 ± 3.775	9.010 ± 3.237	17.83 ± 6.741	6.135 ± 2.628	18.38 ± 5.644
**Glutamine**	136.4 ± 16.95	58.41 ± 14.36 a**	77.76 ± 14.39 a**	113.3 ± 16.67 bc*	58.74 ± 10.18 d*	72.11 ± 18.05 d**
**Glutamate**	3.883 ± 0.6326	1.061 ± 0.2552	1.931 ± 0.4517	2.773 ± 0.8548	1.214 ± 0.1813	2.033 ± 0.7631
**GABA**	283.5 ± 59.00	143.0 ± 24.02 a**	152.3 ± 34.06 a*	347.8 ± 80.01 bc*	142.0 ± 29.95 d*	278.7 ± 67.55 b*
**GABARA**	9.912 ± 1.587	1.752 ± 0.8360 a**	3.096 ± 0.9080 a**	6.479 ± 2.550 bd*	1.324 ± 0.7396 a*	4.493 ± 2.594 a*

(a) Control vs. all groups, (b) PPA vs. all groups, (c) PPA + vs. therapeutic group, (d) PPA + BIF vs. PPA + LAC and PPA + BIF vs. PPA + MIX, and (e) PPA + LAC vs. PPA + MIX. (*) The mean difference is significant at *p* ˂ 0.001, and (**) the mean difference is significant at *p* ˂ 0.0001.

**Table 2:** Means ± SD of all the measured variables. Ratios of (I) GABA/GABARA, (II) GABA/glutamate, and (III) glutamine/glutamate in the brain homogenates of the six studied groups.

	Control	PPA	PPA+	PPA + BIF	PPA + LAC	PPA + MIX
**GABA/GABAR_A_**	25.32 ± 7.092	11.32 ± 5.397 a*	12.64 ± 10.00 a*	13.05 ± 8.337 a*	11.21 ± 3.899 a*	10.59 ± 5.425 a*
**GABA/Glutamate**	83.27 ± 26.58	47.92 ± 19.39	64.26 ± 67.62	81.82 ± 28.73	61.63 ± 40.50	83.06 ± 44.28
**Glutamine/glutamate**	25.00 ± 9.945	16.18 ± 7.730	28.72 ± 13.32	30.90 ± 9.489	25.38 ± 11.18	39.86 ± 12.84 b*

(a) Control vs. all groups and (b) PPA vs. all groups. (*) The mean difference is significant at *p* ˂ 0.001.

**Table 3:** Means ± SD of the gene expression of GABAR_A_, GABAR_B_, and GABAR_G_ selected subunits in the brain homogenates of male western albino young rats in all groups.

	Control	PPA	PPA+	PPA + BIF	PPA + LAC	PPA + MIX
**GABAR_A_1**	1 ± 0.409	0.0716 ± 0.0089 *	1.878 ± 0.086 *	2.493 ± 0.291 **	2.049 ± 0.0789 *	0.0371 ± 0.00073 **
**GABAR_A_2**	1 ± 0.162	0.0116 ± 0.007 **	0.924 ± 0.056401 *	1.574 ± 0.336 *	0.310 ± 0.044 *	0.0259 ± 0.0026 **
**GABAR_A_3**	1 ± 0.027	0.00036 ± 5.18046 × 10^−5^ **	0.648 ± 0.024 **	1.132 ± 0.119 **	0.256 ± 0.0213 **	0.00059 ± 0.00015 **
**GABAR_A_5**	1 ± 0.108	0.0070 ± 0.0011 **	0.564 ± 0.030 **	0.683 ± 0.038 **	0.142 ± 0.004 **	0.019 ± 0.002 **
**GABAR_B_2**	1 ± 0.0195	0.066 ± 0.0137 *	1.076 ± 0.135 *	1.608 ± 0.269 *	0.677 ± 0.662	0.038 ± 0.00196 **
**GABAR_B_3**	1 ± 0.158	0.00025 ± 0.00011 *	1.089 ± 0.0064 *	2.099 ± 0.215 **	0.561 ± 0.087 *	6.81 × 10^−5^ ± 8.92 × 10^−5^ *
**GABAR_G_2**	1 ± 0.199	0.0410 ± 0.00322 *	0.611 ± 0.028 *	1.181 ± 0.1609 *	0.2875 ± 0.0266 *	0.01715 ± 0.0017 *

(*) The mean difference is significant at *p* ˂ 0.001. (**) The mean difference is significant at *p* ˂ 0.0001.

**Table 4:** Estimated changes in microorganisms in all groups. MCA—MacConkey agar; NA—nutrient agar; MHA—Mueller Hinton agar; blood agar. ((-) No growth, (+) weak growth, (++) medium growth, and (+++) strong growth).

Isolated Organisms	Media and Incubation Conditions	Control	PPA+	PPA + BIF	PPA + LAC	PPA + MIX
Enterobacteriaceae (Gram-negative rod, lactose fermenters)	MCA/aerobic 37 °C/24 h	+	-	++	+++	++
*Staphylococcus* and/or Bacilli (Gram-positive cocci/rod or Gram-negative rod)	NA/aerobic 37 °C/24 h	-	-	+++	+	++
*Moraxella* spp.	MHA/aerobic 37 °C/24 h	++	+	++	++	++
Gram-negative						
Gram-positive/Gram-negative rod and positive cocci	Blood/aerobic 37 °C/24 h	-	++	++	-	+

## Data Availability

The datasets and generated analyses during this study are available from the corresponding author on reasonable request.
